# HARP: a database of structural impacts of systematic missense mutations in drug targets of *Mycobacterium leprae*

**DOI:** 10.1016/j.csbj.2020.11.013

**Published:** 2020-11-19

**Authors:** Sundeep Chaitanya Vedithi, Sony Malhotra, Marcin J. Skwark, Asma Munir, Marta Acebrón-García-De-Eulate, Vaishali P Waman, Ali Alsulami, David B Ascher, Tom L Blundell

**Affiliations:** a Department of Biochemistry, University of Cambridge, Tennis Court Rd., CB2 1GA, UK; b Department of Biological Sciences, Institute of Structural and Molecular Biology, Birkbeck College, University of London, Bloomsbury, London WC1E 7HX, United Kingdom; c University College London, Institute of Structural and Molecular Biology, Bloomsbury, London, WC1E 6BT, United Kingdom; d Department of Biochemistry and Molecular Biology, Bio21 Institute, University of Melbourne, Parkville, VIC 3052, Australia; e Structural Biology and Bioinformatics, Baker Heart and Diabetes Institute, Melbourne, VIC 3004, Australia

**Keywords:** Drug resistance, Mutations, Protein stability, Interatomic interactions, *Mycobacterium leprae*, Computational saturation mutagenesis

## Abstract

Computational Saturation Mutagenesis is an *in-silico* approach that employs systematic mutagenesis of each amino acid residue in the protein to all other amino acid types, and predicts changes in thermodynamic stability and affinity to the other subunits/protein counterparts, ligands and nucleic acid molecules. The data thus generated are useful in understanding the functional consequences of mutations in antimicrobial resistance phenotypes. In this study, we applied computational saturation mutagenesis to three important drug-targets in *Mycobacterium leprae (M. leprae)* for the drugs dapsone, rifampin and ofloxacin namely Dihydropteroate Synthase (DHPS), RNA Polymerase (RNAP) and DNA Gyrase (GYR), respectively. *M. leprae* causes leprosy and is an obligate intracellular bacillus with limited protein structural information associating mutations with phenotypic resistance outcomes in leprosy. Experimentally solved structures of DHPS, RNAP and GYR of *M. leprae* are not available in the Protein Data Bank, therefore, we modelled the structures of these proteins using template-based comparative modelling and introduced systematic mutations in each model generating 80,902 mutations and mutant structures for all the three proteins. Impacts of mutations on stability and protein-subunit, protein-ligand and protein-nucleic acid affinities were computed using various in-house developed and other published protein stability and affinity prediction software. A consensus impact was estimated for each mutation using qualitative scoring metrics for physicochemical properties and by a categorical grouping of stability and affinity predictions. We developed a web database named HARP (a database of **H**ansen's Disease **A**ntimicrobial **R**esistance **P**rofiles), which is accessible at the URL - **https://harp-leprosy.org** and provides the details to each of these predictions.

## Graphical abstract

**Figure:**
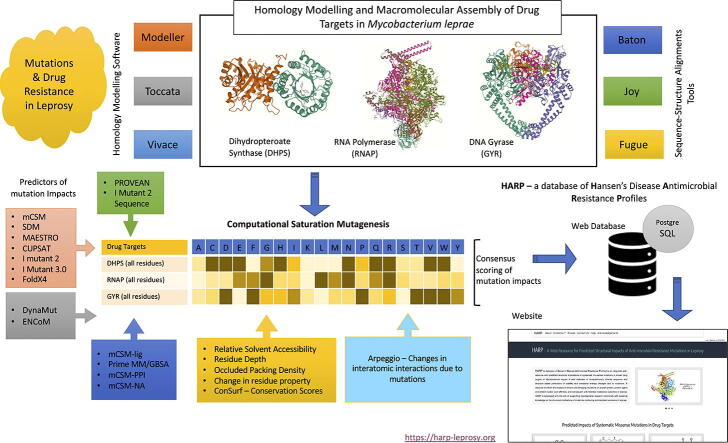

## 1 Introduction

*Mycobacterium leprae* (*M. leprae*) is a pathogenic species of mycobacterium that causes leprosy (also known as Hansen's disease) in tropical countries. Approximately 210,000 new cases of leprosy are reported each year globally [1]. Leprosy causes slowly progressive sensorimotor polyneuropathy [2] in the peripheral nerves leading to permanent nerve damage and deformities. The disease is currently treated by multidrug therapy that includes dapsone, rifampin and clofazimine. Earlier monotherapies with dapsone and rifampin have led to the emergence of resistant strains of *M. leprae* for dapsone in the year 1964 and for rifampin in 1976 [3]. This has led to the introduction of multidrug therapy (MDT) by the World Health Organisation (WHO) in 1983. In the absence of a microbiological propagation media for *M. leprae*, clinical insensitivity to drugs is regarded as a sign of drug-resistance/relapse. Resistance can be noted either during MDT (primary resistance) or after the completion of standard WHO-recommended regimen of MDT (secondary resistance) [4]. *In-vivo* propagation of *M. leprae* in the hind footpads of experimental mice administered with individual drugs of MDT is regarded as a gold-standard method for determining drug resistance [5]; however, this approach is time and labour intensive and is limited to laboratories specialised in animal experiments. As in *Mycobacterium tuberculosis* (*M. tb*), substitution mutations within the drug resistance determining regions (DRDR) of genes that encode drug-targets demonstrate an association with phenotypic resistance outcomes in leprosy [6].

Antimycobacterial drugs interact with specific proteins (drug-targets) in mycobacteria and inhibit/attenuate their function. This interaction is governed by interatomic bonds between the drug molecule and amino acid residues in the active site/drug binding site of the target protein. The occurrence of missense mutations in the bacterial genomes result in amino acid substitutions which disturb these interactions, alter the thermodynamic stability of the protein and affect protein-ligand affinity leading to phenotypic drug resistance, a state in which the bacteria turns insensitive to the drug [7], [8], [9], [10], [11]. Missense mutations are known to alter thermodynamic stability of the proteins [12] leading to either loss or gain in the function of the target protein. They also confer changes in affinity to ligands, nucleic acids, other proteins and small molecules. The methods developed in our group were focused either on using statistical potentials to measure the difference in free energy change between wildtype and mutant structures in folded and unfolded states [13] or machine learning approaches that adopt graph-based signatures derived from interatomic distance matrices between the mutating residue and the residue environment, and the pharmacophoric properties of the mutating residue [14]. Proteins being dynamic molecules, substitution mutations impact molecular motions leading to a change in the flexible conformations and vibrational entropy. Most of the tools that predict thermodynamic stability changes assess the change in a static state [15]. Employing molecular dynamics simulations and normal mode analysis aid accurate assessment of stability changes [16]. However, molecular dynamic simulations are computation and data-intensive and can be complemented to an extent by tools that employ normal mode perturbations [17]. Predicting the consequences of point mutations in leprosy with considerable accuracy and identifying their association with antimicrobial resistance outcomes are essential due to the lack of robust experimental methods of diagnosing resistance.

Computational saturation mutagenesis [18] is an approach that aids in systematically analysing the impacts of all possible substitution mutations at a given residue position in the protein. We have applied this approach earlier to the beta subunit of RNA Polymerase (RNAP) [19]. We have now extended this approach and applied it to the three known drug-targets in *M. leprae,* the Dihydropteroate Synthase (DHPS), RNA Polymerase (RNAP) and DNA Gyrase (GYR) that are the targets of dapsone, rifampin and ofloxacin respectively. Mutations within the DRDR of genes encoding target proteins are known to confer antimicrobial resistance in leprosy [20]. Strains of *M. leprae* that carry these mutations exhibit various levels of resistance, as noted by their response to different concentrations of drugs in the murine model of drug sensitivity assessment [21]. We modelled the structures of the three drug-targets described above using template-based modelling and introduced systematic mutations in each structure, generating 80,902 mutant models. Consequences of mutations on protein stability and affinity to other subunits in the oligomeric complexes, nucleic acids and ligands were calculated using a suite of software tools [19]. A consensus impact was estimated for each mutation and represented in a publicly available HARP database (URL: https://harp-leprosy.org) with features to interactively visualize the wildtype and the mutant models*.* This resource can provide comprehensive structural insights into the potential implications of missense mutations in antimicrobial-resistant leprosy. This database also enables visualizing sites on the drug-target proteins that are least impacted by any mutations, and these can be explored for structure-guided drug discovery.

## 2 Materials and methods

### 2.1 Comparative modelling of DHPS, RNAP and GYR

We performed comparative 3D modelling of DHPS, RNAP and GYR using Modeller 9.24 [22]. The models of DHPS and RNAP were built as reported by us earlier [12], [23]. The model of DNA-gyrase (GYR) was built using PDB id: 5BS8 (Crystal structure of a topoisomerase II complex of *M tb at* 2.40 *Å resolution* [24]) as a template. This heterotetrameric protein (GyrA2, GyrB2) is comprised of four chains (A, B, C, D), which are encoded by *gyrA* (ML0006) (homodimer of chains A and C) and *gyrB* (ML0005) (homodimer of chains B and D). The ML0006 has an identity of 91% with its *M. tb* (strain H37Rv) homologue Rv0006, and ML0005 has an identity of 88% with Rv0005. The chain A in *M. leprae* has an intein region [25] stretching from residue positions 131–500. This sequence has been removed before modelling. The modelled region of chain A corresponds to sequence numbers 16–130 and 551–921. The chain B is modelled from residue numbers 440–678. Quality of the built models was estimated using MolProbity [26], a structure validation web service that provides a comprehensive evaluation of the model quality at both global and local levels for proteins and nucleic acids. The MolProbity score resembles the X-ray crystallographic resolution of the protein structures. Molprobity score for DHPS is 1.34 at 98th percentile (100th percentile is the best among structures of comparable resolution), for RNAP, it is 1.48 at 95th percentile, and for GYR, the score is 0.86 at 100th percentile indicating that these models are of optimal quality for further analysis. The ligands, dapsone for DHPS, rifampin for RNAP and ofloxacin for GYR, were modelled in their respective binding sites. Dapsone and ofloxacin were docked into DHPS and GYR binding sites respectively by molecular docking (using Glide XP module [27] from Schrodinger Suite 2019-4). Rifampin was introduced by the superimposition of the model with the template structure (PDB id: 5UHC). The models were visualized using UCSF Chimera [28].

### 2.2 Residue properties and conservation scores

The residue properties, conservation score of the wildtype residue, change in secondary structure, residue depth, relative solvent accessibility and residue occluded packing density (OSP) were calculated using ConSurf [29] and SDM [13]. Additionally, the distances of each residue from the closest ligand, protein interface and nucleic acids were also calculated using inhouse written Bioperl scripts. We have used these properties to classify the impacts of substitution mutations on the residue environment.

### 2.3 Prediction of changes in thermodynamic stability

To predict thermodynamic stability changes due to mutations based on the structural properties, we employed mCSM [14], SDM, MAESTRO [30], CUPSAT [31], IMutant 2.0 structure [32] and IMutant 3.0 [33]. For the sequence-based prediction of stability changes, we used PROVEAN [34]and IMutant 2.0 sequence [32]. To understand the impacts of mutations on the vibrational entropy and protein motions, we employed DynaMut [35], ENCoM [36] and FoldX4 [37]. Gibbs free energy changes (ΔΔG in kcal/mol) -were calculated using standalone versions of these online tools. A brief description of each of these tools is provided in Table 1.

**Table 1:** Tools used in predicting protein stability and affinity changes resulting rom mutations.

Tool [reference]	Description	Website	Input
Protein Stability Changes:			
mCSM [14]	Predicts the effect of mutations in proteins using graph-based signatures	http://biosig.unimelb.edu.au/mcsm/	Drug-target model (PDB file) and list of mutations on the web interface
SDM [13]	Predicts the stability of proteins due to mutations using residue environment-specific substitution matrices	http://marid.bioc.cam.ac.uk/sdm2/	Drug-target model (PDB file) and list of mutations submitted to the local version of the SDM software
MAESTRO [30]	A multiagent machine learning approach to predict free energy changes due to mutations	https://pbwww.che.sbg.ac.at/?page_id=416	Drug-target model (PDB file) and list of mutations on a Linux shell interface with the local version of the software
CUPSAT [31]	This program uses structural environment-specific atom potentials and torsion angle potentials to predict ΔΔG	http://cupsat.tu-bs.de/	Drug-target model (PDB file) and list of mutations on the web interface.
I Mutant 2.0 Structure [32]	A support vector machine-based tool to conduct a regression estimate of the ΔΔG values using experimental thermodynamic data	https://folding.biofold.org/i-mutant/i-mutant2.0.html	Drug-target model (PDB file) and list of mutations on a Linux shell interface with the local version of the software
I Mutant 3.0 Structure [33]	A three-state predictor of protein stability changes that classify impacts as destabilizing, stabilizing and neutral mutations	http://gpcr2.biocomp.unibo.it/cgi/predictors/I-Mutant3.0/I-Mutant3.0.cgi	Drug-target model (PDB file) and list of mutations on a Linux shell interface with the local version of the software
PROVEAN [34]	A software tool to predict the impact of amino acid substitution on the biological function of the protein	http://provean.jcvi.org/index.php	Drug-target model (PDB file) and list of mutations on the web interface.
IMutant 2.0 Sequence [32]	A support vector machine-based tool to conduct regression estimates of the ΔΔG values using amino acid substitution data	https://folding.biofold.org/i-mutant/i-mutant2.0.html	Drug-target model (PDB file) and list of mutations on a Linux shell interface with the local version of the software
DynaMut [35]	A tool to predict protein stability changes upon mutations using normal mode analysis	http://biosig.unimelb.edu.au/dynamut/	Drug-target model (PDB file) and list of mutations on the web interface.
ENCoM [36]	A coarse-grained normal mode analysis method used to predict the effects of single point mutations on protein dynamics and thermostability resulting from vibrational entropy changes	https://github.com/NRGlab/ENCoM	Drug-target model (PDB file) and list of mutations on a Linux shell interface with the local version of the software
FoldX [37]	This program employs an empirical force field for the rapid evaluation of the effect of mutations on the stability, folding and dynamics of proteins and nucleic acids	http://foldxsuite.crg.eu/command/BuildModel	Drug-target model (PDB file) and list of mutations on a Linux shell interface with the local version of the software

**Protein-Ligand Affinity:**			
mCSM-lig [38]	A tool to quantifying the effects of mutations on protein-ligand affinity in genetic diseases and the emergence of drug resistance	http://biosig.unimelb.edu.au/mcsm_lig/	Drug-target model (PDB file) and list of mutations on the web interface.
Prime MM/GBSA [39]	A software tool that generates energy properties of the ligand, receptor and the complex, and enable calculation of changes in the mutants	https://www.schrodinger.com/kb/1484	Drug-target model (PDB file) and list of mutations on the local Maestro GUI.

**Protein Nucleic Acid Affinity:**			
mCSM-NA [40]	A program to predict the changes in protein-nucleic affinity due to mutations	http://biosig.unimelb.edu.au/mcsm_na/	Drug-target model (PDB file) and list of mutations on the web interface.

**Protein-protein Affinity:**			
mCSM-PPI [14]	A program to predict the changes in protein-protein affinity due to mutations	http://biosig.unimelb.edu.au/mcsm/protein_protein	Drug-target model (PDB file) and list of mutations on the web interface.

**Residue Conservation:**			
ConSurf [29]	Predict evolutionary conservation of amino/nucleic acid positions in a protein/DNA/RNA molecule based on the phylogenetic relations between homologous sequences	https://consurf.tau.ac.il/	Drug-target model (PDB file)

**Interatomic Interactions:**			
Arpeggio [41]	A webserver for calculating interatomic interactions in protein structures.	http://biosig.unimelb.edu.au/arpeggioweb/	Drug-target model (PDB file) and the residue selection.

### 2.4 Prediction of protein-protein, protein-ligand and protein-nucleic affinity changes due to mutations

Missense mutations impact not only the thermodynamic stability of the proteins but also protein-ligand, protein-nucleic acid and protein-protein affinities. We used mCSM-lig and Prime MM/GBSA programs to measure the impacts of the mutations on protein-ligand affinity. For RNAP, mCSM-lig was used to predict the impacts of systematic mutations for residues within 5 Å of rifampin. For DHPS and GYR, we used Prime MM/GBSA to estimate the change in affinity to dapsone and ofloxacin, respectively. The changes were computed for all residues that are within 5 Å in distance to the ligand. mCSM-lig calculates the log change in affinity for the ligand between the wildtype and mutant structures. MM/GBSA is an approach that combines molecular mechanics energies with generalised Born and surface area continuum solvation methods to estimate the free energy of binding of the ligands to protein macromolecules. We calculated MM/GBSA values for the wildtype and the mutant models. To estimate the change in protein-protein affinity due to mutations, we employed mCSM-PPI and for change in affinity to nucleic acids, mCSM-NA was used.

### 2.5 Interatomic interactions

Further, we generated mutant structures for all 80,902 mutations in DHPS, RNAP and GYR using Modeller v9.24. We then calculated interatomic interactions of the wildtype as well as the mutant residues with the surrounding residue environment using Arpeggio, an in-house developed tool for calculating interactions based on interatomic and interresidue distances.

### 2.6 Consensus scoring of mutation impacts

We adopted a qualitative scoring approach to measuring the consensus impact of the mutation on drug-target stability and its affinity to ligands, nucleic acids and other protein subunits. The changes in residue properties and the residue environment due to mutations, are classified to have an either high or low impact on the protein structure as shown in Table 2.

**Table 2:** Properties of the wildtype and the mutant residues, and their impact on protein structure

Property	Outcome
Residue properties of wildtype and mutant are the same (e.g., aliphatic to aliphatic substitution)	Low Impact
Change in residue property of the mutant (e.g., aliphatic to aromatic substitution)	High Impact
Conservation score > 0(variable residue) (as measured by ConSurf)	Low Impact
Conservation score < 0 (conserved residue)	High Impact
Interface Residue = No (more than 5 Å from the subunit interface)	Low Impact
If the mutating residue is an interface residue (<5 Å from the subunit interface)	High Impact
No change in secondary structure due to mutation (identified using SDM2)	Low Impact
Change in secondary structure due to mutation	High Impact
If the distance of the mutating residue from the ligand is < 5 Å	High Impact
If the distance of the mutating residue from the ligand is >5 Å	Low Impact
If the distance of the mutating residue from the nucleic acid is < 5 Å	High Impact
If the distance of the mutating residue from the nucleic acid is >5 Å	Low Impact

**Fig. 1:**
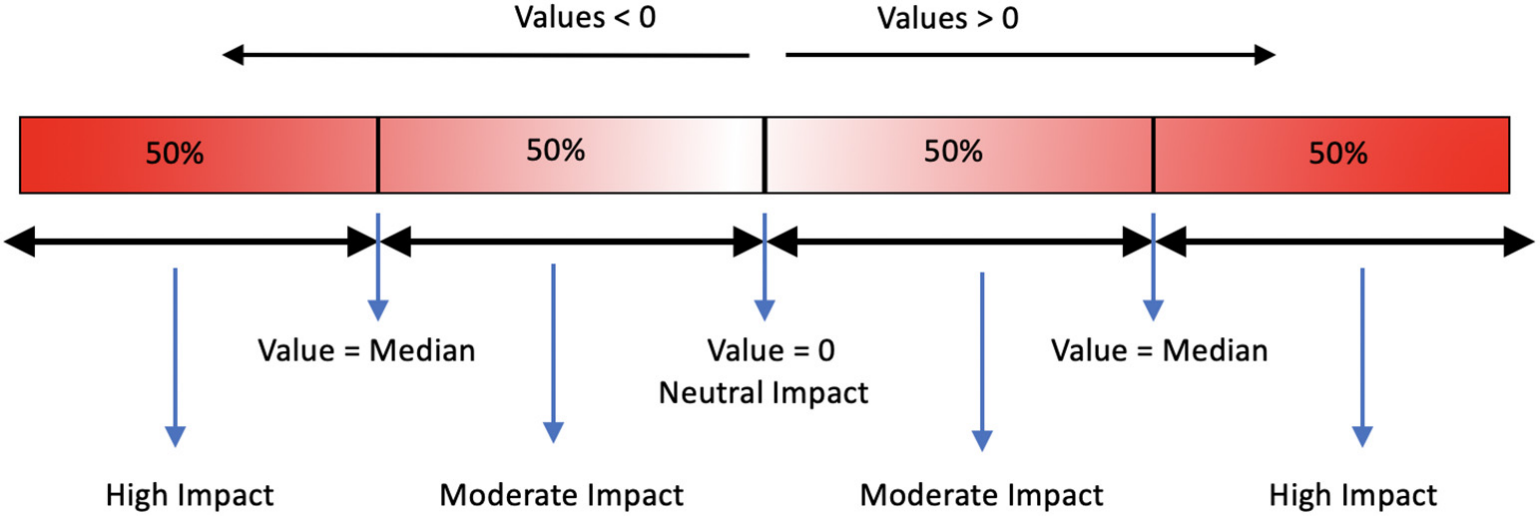
Attribution of categorical variables to continuous data (value) for the differences in relative solvent accessibility, residue depth and OSP for mutations at each residue position.

The differences in residue solvent accessibility, residue depth and OSP between wildtype and mutant residues are calculated using SDM. The values for each mutation above and below zero are split at the median, and the corresponding categorical variables are assigned as shown in Fig. 1.

For stability and affinity predictions using mCSM, mCSM-PPI, mCSM-lig, Prime MM/GBSA, mCSM-NA, SDM, MAESTRO, IMutant 3, FoldX4 and DynaMut, a similar approach to that described in Fig. 1 was adopted with zero as the median value denoted as neutral. The values below zero are categorised as highly destabilising (less than the median) and destabilising (less than zero and greater than the median) respectively. The values above zero are categorised as highly stabilising (above the median) and stabilising (greater than zero and less than the median) respectively. For the tools, PROVEAN (output = Neutral, Deleterious), CUPSAT (output = Stabilising, Neutral, Destabilising), CUPSAT Torsion (output = Unfavourable, Neutral, Favourable), IMutant-2 structure (output = Decreased Stability, Increased stability), IMutant-2 Sequence (output = Decreased stability, Increased stability) and EnCOM (output = Increased Molecular Flexibility, Decreased Molecular Flexibility), the corresponding output terms in the brackets were used as provided by the software. In total, there are 22 estimates from which the overall score was calculated.

From all the program outputs, the destabilising terms listed are “highly destabilising”, “destabilising”, “decreased stability”, “deleterious”, “increased molecular flexibility”, “unfavourable”, “reduced Stability”, “high impact” and “moderate impact”. The neutral and stabilising terms are “highly stabilising ”, “stabilising ”, “increased stability”, “neutral”, “decreased molecular flexibility”, “favourable”, “increased stability”, and “low impact”. These terms are unitised, and overall impact of a mutation is scored as follows:

$$\mathrm{Overall}\;\mathrm{score}=\left(\mathrm{sum}\;\mathrm{of}\;\mathrm{the}\;\mathrm{destabilising}\;\mathrm{terms}\right)\;-\;\left(\mathrm{sum}\;\mathrm{of}\;\mathrm{the}\;\mathrm{stabilising}\;\mathrm{terms}\right).$$

Scores for all mutations in each drug-target are then categorised, as shown in Fig. 1. The corresponding categorical attribute for each mutation is considered as the overall impact of the mutation on the structure of the drug-target.

### 2.7 Web server

After collecting and analyzing the predictions from all the tools listed in Table 1, we developed a PostgreSQL database using Flask SQLAlchemy framework. This web-database enables the users to query any possible mutation in all the three drug-targets of *M. leprae*, the DHPS, RNAP and GYR and obtain predictions from all the tools stated in Table 1 and also provide options to download wildtype and mutant models. An interactive viewer powered by Molstar [42] enables the users to view the models interactively and recognise the changes in interatomic interactions of the wildtype and the mutant residues within their residue environments. This versatile web-interface is developed with modern web standards and is made available on the web.

### 2.8 Data curation

Experimentally identified mutations were manually collected and collated from the published literature using the search terms/phrases: “mutations”, “drug-resistance”, “leprosy”, “*Mycobacterium leprae”*, “leprosy relapse”, “dapsone resistance”, “rifampicin resistance”, “ofloxacin resistance” and “drug resistance determining regions” in various combinations on search engines such as PubMed, Google Scholar and Google search. Only original articles and case reports that detected mutations in patient samples were included in the study. Mutations noted for dapsone, rifampin and ofloxacin from these published articles demonstrated varying levels of association with the clinical insensitivity to corresponding drugs; however, only those mutations reported by the WHO sentinel surveillance network for drug resistance in leprosy, are known to be experimentally validated in the mouse footpad models [3]. As this study is aimed at deciphering the structural impacts of missense mutations, indels and synonymous mutations were excluded from the data.

## 3 Results

### 3.1 The HARP database

**Fig. 2:**
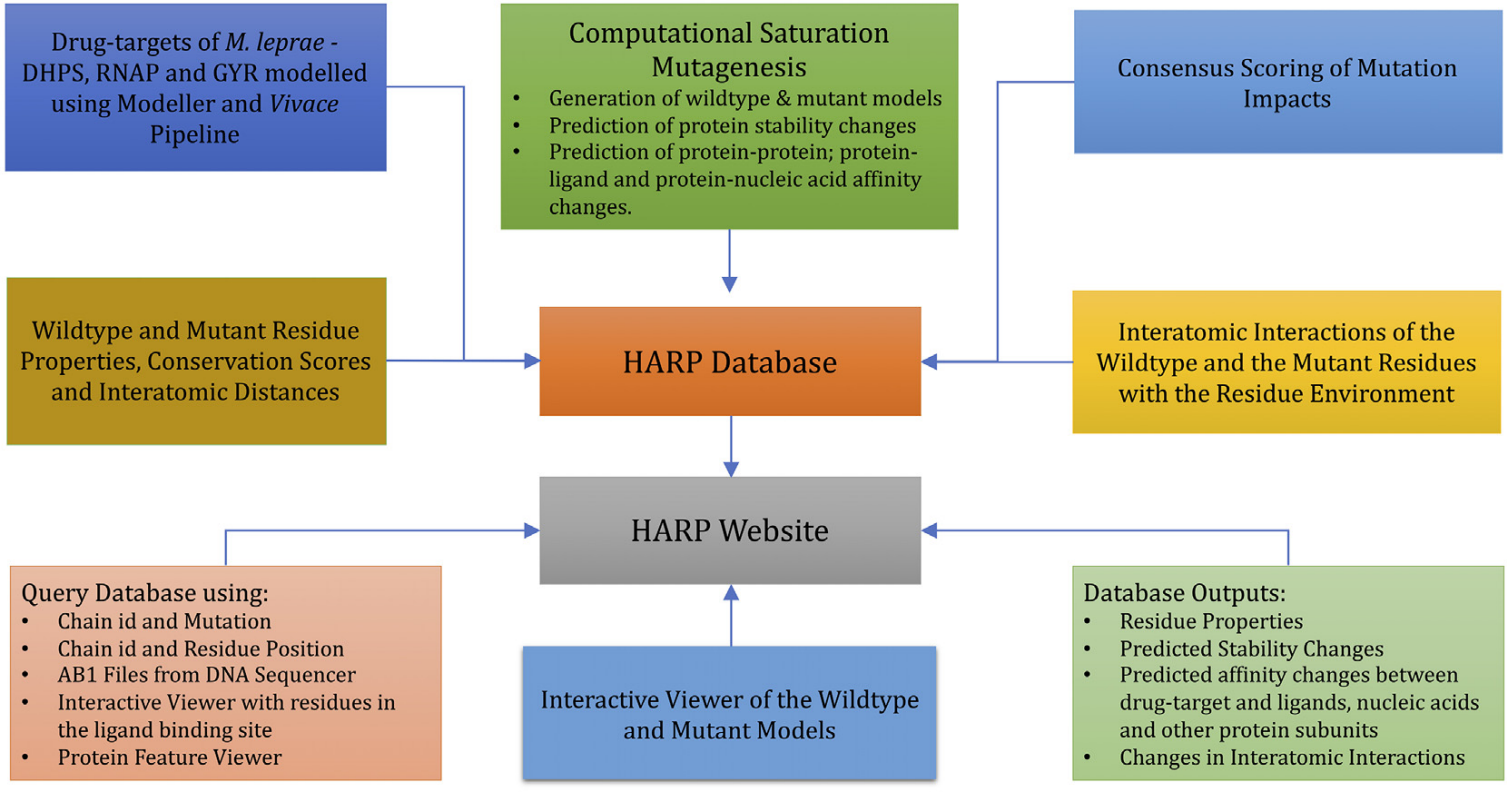
An overview of the HARP database. (DHPS - Dihydropteroate Synthase, RNAP - RNA Polymerase and GYR - DNA Gyrase).

The HARP database (**H**ansen's disease **A**ntimicrobial **R**esistance **P**rofiles) is a collection of drug-target stability and affinity changes due to mutations predicted using structure, sequence and vibrational entropy features. An overview of the HARP database and the web-interface is shown in Fig. 2.

### 3.2 The HARP web interface

#### 3.2.1 Querying mutations

**Fig. 3:**
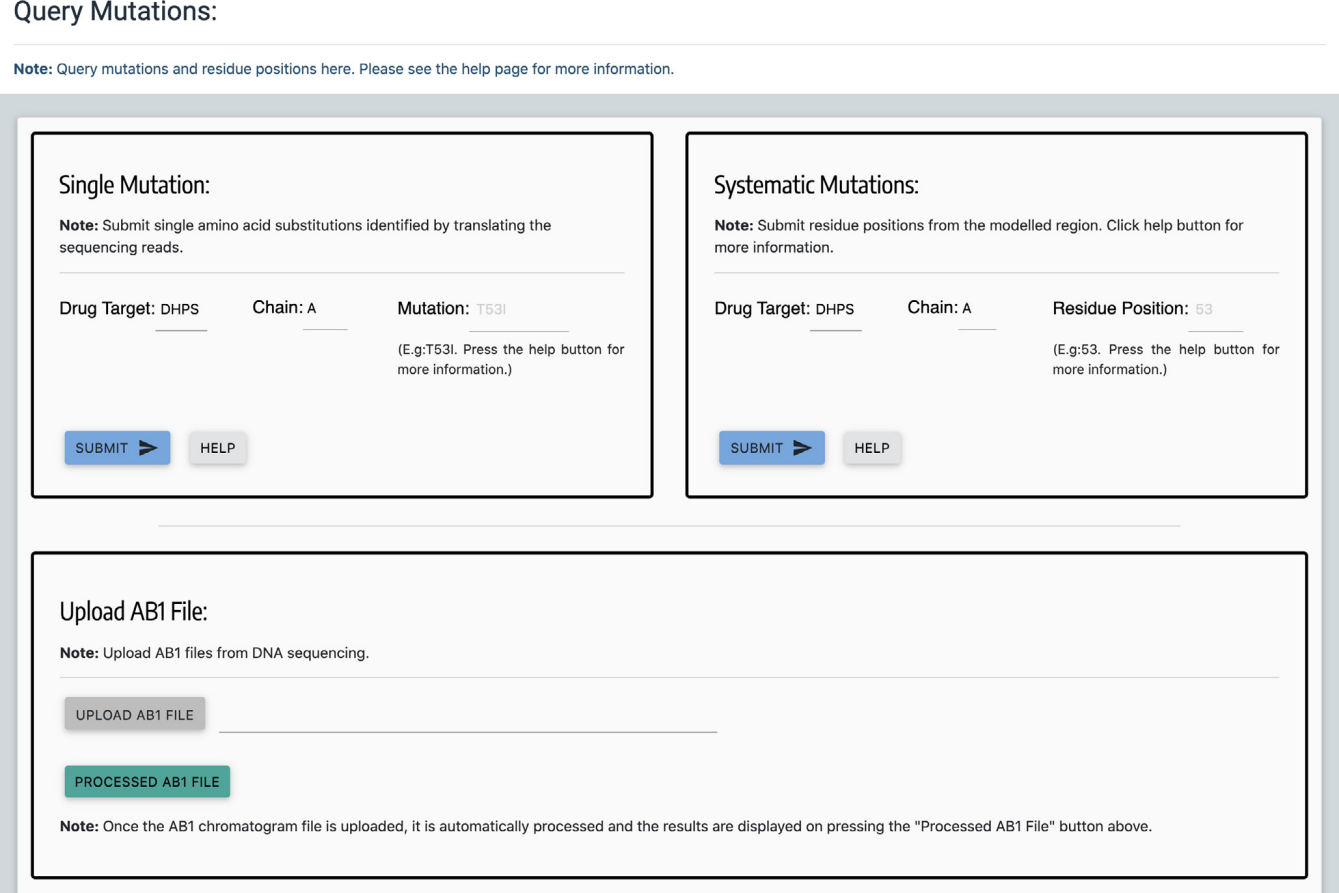
Web page for querying mutations. Users can query single mutations using chain id and mutation in the “Single Mutation” form or systematic mutations (all 19 possibilities) using residue number in the “Systematic Mutations” form. Additionally, users can upload AB1 Chromatogram files and obtain the BlastX results.

One of the important outcomes of this study is the development of HARP web database. HARP embodies systematic computational saturation mutagenesis of all the three known drug-target proteins in *M. leprae* namely DHPS, RNAP and GYR with predicted impacts resulting from mutations on thermodynamic stability and affinity to other proteins, ligands and nucleic acids. It enables the mycobacterial research community to harness the knowledge related to structural impacts of any possible mutations in these drug targets that confer antimicrobial resistance in leprosy. User can query mutations using buttons with drug names on the home page or from the “Mutations” link on the top navigation bar. On the mutations page (Fig. 3), users can query mutations either by submitting the protein chain id and the mutation (single mutation) or the chain id and the residue position (systematic mutations). For diagnosis of drug resistance in leprosy, the DRDRs of the drug-target coding genes are amplified and sequenced. HARP enables users to process the AB1 chromatogram files from the DNA sequencer and help detect mutations by performing translated nucleotide blast (BlastX) [43] on the protein sequence. Additionally, there is a 3D viewer powered by NGL [44] to visualise the residues that interact with the ligand in each drug-target using interactive mouse controls. Finally, there is a protein feature viewer [45] to visualise the protein sequence and other sequence-derived properties.

#### 3.2.2 Residue properties and predicted stability changes

**Fig. 4:**
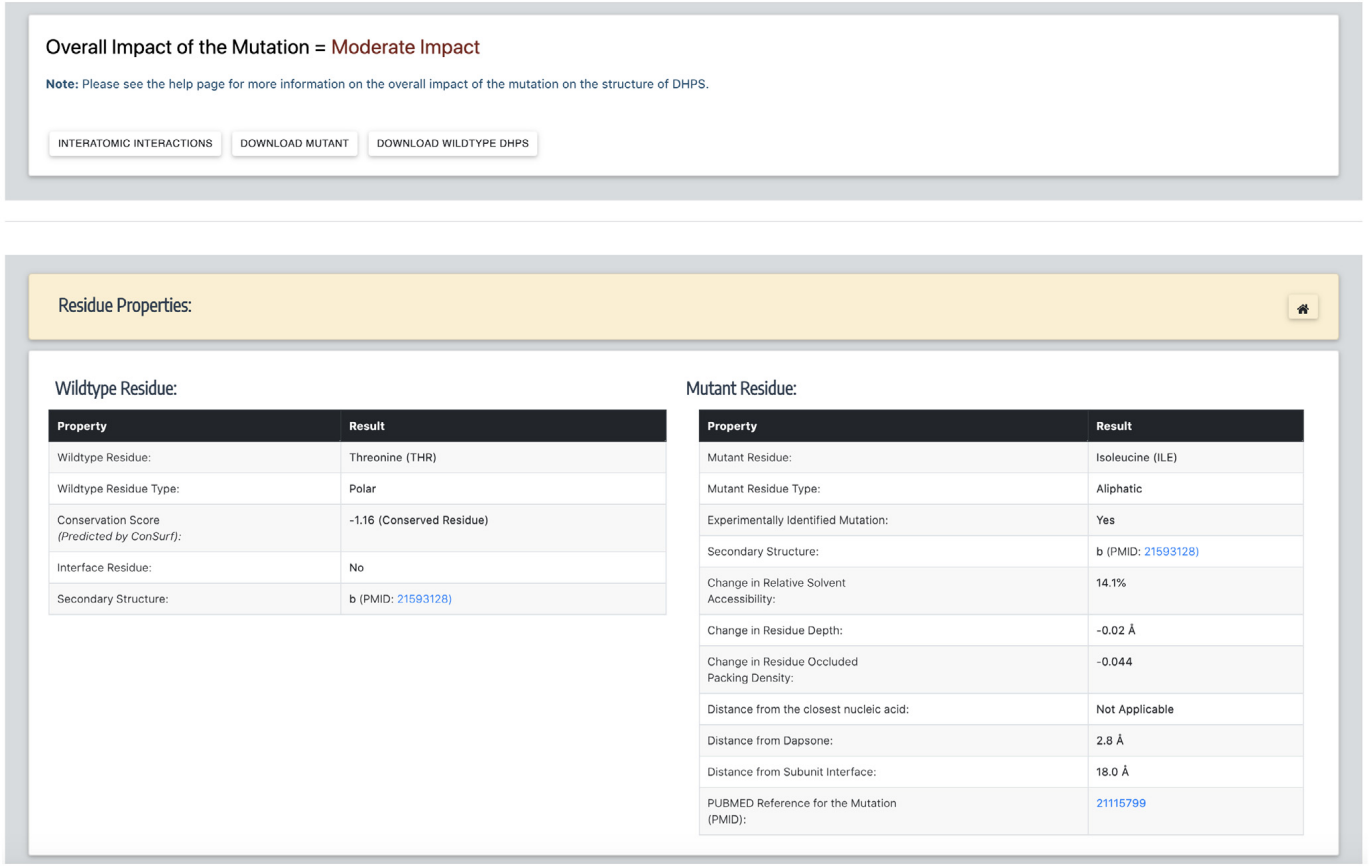
The results page, after submitting chain id and mutation in the “Single Mutations” form. This page provides the user with an option to download the models and also visualize the structure by following appropriate links. It lists all the predictions for stability and affinity changes due to mutations.

Once the chain id and mutation are submitted, the overall impact of the mutation, options to download wildtype and mutant models, wildtype and mutant residue properties, structure-based changes in protein stability, sequence-based stability and vibrational entropy changes can be viewed on the results page (Fig. 4). For the systematic mutations form, once the residue position is submitted, predictions for all 19 possible mutations at the queried residue position can be viewed in the form of downloadable tables. To obtain comprehensive information about a specific mutation, the user can copy the mutation into the “Single Mutation” form to download wildtype and the mutant models or interactively visualize the structures by clicking on the “Interatomic Interactions” link. Under the mutant properties, there is an option to look at the associated publication if the specific mutation is clinically identified in drug-resistant leprosy patients.

#### 3.2.3 Interatomic interactions and 3D visualization of the models

**Fig. 5:**
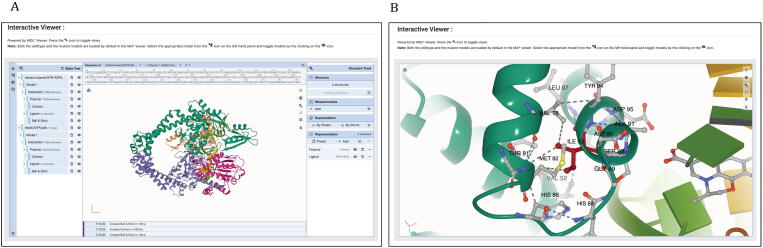
A: Interactive viewer on the interactions page enables the user to view models in various representations and recognize changes in interatomic interactions in the wildtype and the mutant models. B: Residue isoleucine at position 93 was focused in the same viewer with left and right panels hidden. The viewer enables visualizing interatomic interactions of the selected residues in both wildtype and mutant models. The grey dotted lines indicate hydrophobic bonds and blue dotted lines indicate hydrogen bonds. (For interpretation of the references to color in this figure legend, the reader is referred to the web version of this article.)

From the results page, the user can click on the link “Interatomic Interactions” that redirect to the interactions page which has Molstar viewer to visualise the models in 3D. In the Molstar viewer, both the wildtype and the mutant models are loaded by default. User can toggle the views between both the models by clicking appropriate icons as shown in the help notes (Fig. 5). Sequence viewer on the top of the visualiser enables users to select the appropriate residue and view the interatomic interactions that the residue makes with the surrounding residue environment in the protein. Under the “Representation” menu on the right-hand panel, the user can select the whole model or a part of it and change the representations, view different types of interatomic interactions (by clicking on the settings button) and edit the labels. These are few among many features that this visualiser presents to the user, and the user can explore these features using help icons in the viewer.

Additionally, we used the Arpeggio program to calculate interatomic interactions of the wildtype and the mutant residues with the residue environment. The user can recognize the differences in interactions by viewing the tables on the webpage or specifically with atom names by downloading the comma-separated version (csv) files.

#### 3.2.4 Browsing HARP database

The database also provides features for combinatorial browsing and filtering of mutations based on the predicted impacts for each drug-target. User can select appropriate items from the drop-down lists on the “Browse” page and filter the mutations. As DHPS has no nucleic acid molecules in its structure, the drop-down item for protein-nucleic acid affinity for DHPS is “Not-Applicable” by default. Users can change this to other options when browsing mutations in RNAP and GYR. As both RNAP and GYR models have nucleic acids in them, changing the default option of “Not-Applicable” to others in the drop-down menu corresponding to protein-nucleic acid affinity is essential to filter the mutations in RNAP and GYR.

### 3.3 DHPS (Dihydropteroate synthase)

Dihydropteroate synthase catalyses the condensation reaction of 6-hydroxymethyl-7,8-dihydropteridine pyrophosphate to para-aminobenzoic acid to form 7,8-dihydropteroate. The final product in this three-step reaction yields 7,8-dihydrofolate, an intermediate in the folic acid biosynthesis by *M. leprae.* Dapsone competes with para-aminobenzoic acid and inhibits the function of DHPS, leading to the interruption of folic acid biosynthesis. The homodimer of DHPS of *M. leprae* was modelled using its homologue in *M. tb* as a template (PDB id: 1EYE), with the sequence identity of 77%. Dapsone was docked into the binding site as described in [23] and the impacts of saturated mutations were analysed. The structure is modelled from residues 5 to 278 corresponding to the template. A total of 5206 mutations were analysed from 274 residues in chain A. Mutant models were generated using '*mutate_model'* script in Modeller 9.24. The predicted impacts for clinically identified mutations were shown in Table 3.

**Table 3:** Predicted stability and affinity change for clinically identified mutations in dapsone resistant *M. leprae* strains.

Mutations in chain A of DHPS	mCSM (ΔΔG in kcal/mol)	Prime MM/GBSA* (ΔΔG in kcal/mol)	mCSM-ppi (ΔΔG in kcal/mol)	DynaMut (ΔΔG in kcal/mol)	Overall Impact	Reference
V39I	−0.61	NA	−0.83	0.75	Moderate Impact	[46]
V48G	−2.78	NA	−0.75	−10.00	High Impact	[47]
V48A	−2.24	NA	−0.86	−6.02	High Impact	[47]
V48L	−0.72	NA	−1.23	0.44	Moderate Impact	[47]
V48I	−0.72	NA	−1.23	1.35	Moderate Impact	[47]
V48F	−1.42	NA	−0.87	−1.26	Moderate Impact	[47]
V48D	−2.71	NA	−0.91	−8.69	Moderate Impact	[47]
T53A	−0.59	−3.85	−0.09	0.11	High Impact	[47]
T53S	−0.40	−2.69	−0.24	−1.09	High Impact	[47]
T53V	−0.43	−3.69	−0.04	1.12	High Impact	[48]
T53I	−0.40	0.20	−0.01	2.58	Moderate Impact	[47]
T53P	−0.43	−1.30	−0.04	1.05	High Impact	[47]
T53N	−0.21	−2.89	−0.27	0.87	High Impact	[47]
R54G	−0.52	−9.12	−0.66	−0.11	High Impact	[47]
R54W	−0.26	−26.83	−0.85	−0.25	High Impact	[47]
P55A	−0.48	−2.78	0.03	0.14	Moderate Impact	[47]
P55T	−0.44	−1.17	0.38	−0.14	High Impact	[47]
P55S	−0.44	−3.51	0.34	0.14	Moderate Impact	[47]
P55L	−0.28	−2.77	0.18	1.56	Moderate Impact	[47]
P55H	−0.21	−2.74	0.88	0.50	Moderate Impact	[47]
P55R	0.17	−4.06	0.11	−0.32	Moderate Impact	[47]
T88P	−0.35	NA	−0.20	−1.84	High Impact	[49]
D91H	0.04	NA	0.79	−0.01	Moderate Impact	[49]
R94W	−0.21	NA	−0.83	2.18	Moderate Impact	[49]

*NA = Not applicable. For Prime MM/GBSA, NA indicates that the residue is more than 5 Å from dapsone.

Of all the predictors listed in Table 1, only mCSM (change in protein stability), mCSM-PPI (change in stability of the interfacial residues), Prime MM/GBSA (for Ligand affinity), DynaMut (change instability using Normal mode analysis) and FoldX (an empirical force field to determine the change in stability in folded and unfolded states) are shown in Table 3 as they represent diversity and the types of tools used in calculating overall impacts. In the column for Prime MM/GBSA, a value of NA indicates ‘not applicable’ as the residue is located at a distance of more than 5 Å away from dapsone.

**Fig. 6:**
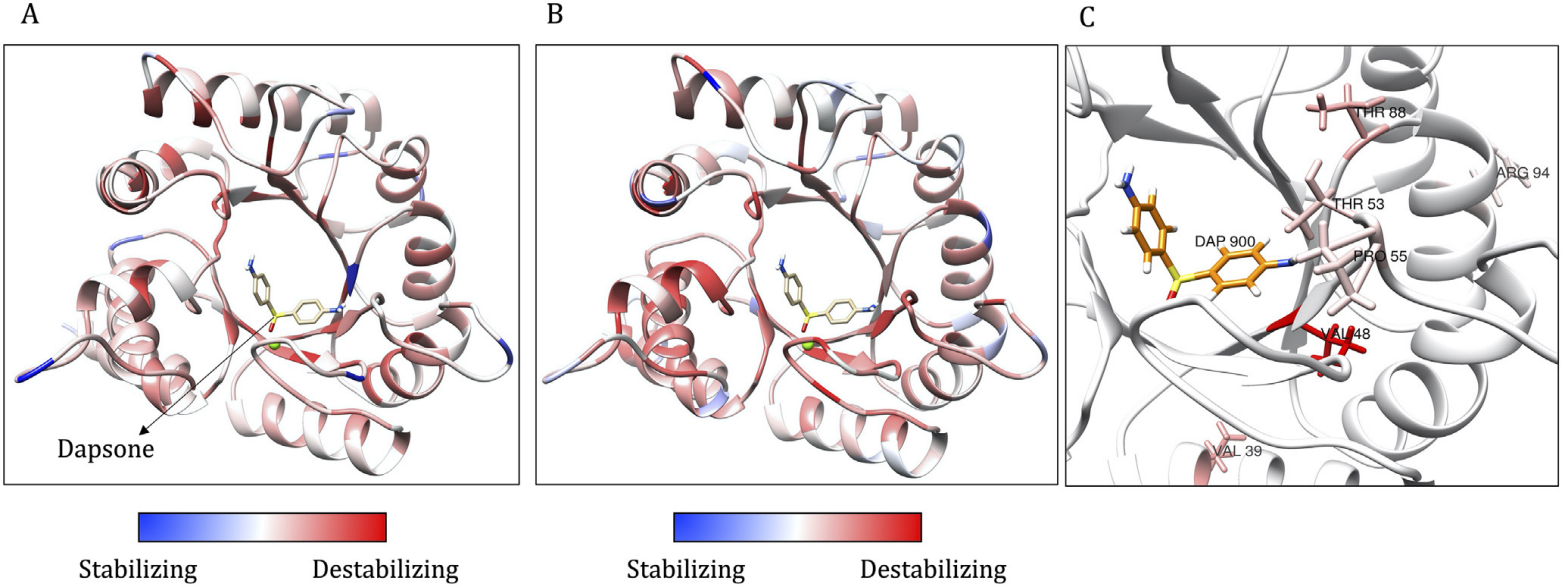
Monomeric model of *M. leprae* DHPS. A: The average destabilizing effects for all possible mutations at each residue position as estimated by mCSM is depicted on the model. The values are color-coded as shown in the scales. B: The average scores of the mutation impacts at each residue position (calculated as described in the methods section) and depicted on the model. C: Average stability changes predicted by mCSM at residue positions where mutations were identified clinically in dapsone-resistant leprosy cases. Mutations were noted at eight residue positions as shown in Table 3. These positions were color-coded based on the average stability changes for any mutation from red (highly destabilizing) to blue (stabilizing). (For interpretation of the references to color in this figure legend, the reader is referred to the web version of this article.)

From the saturation mutagenesis, the average stability changes calculated by mCSM for all possible mutations at each residue position are depicted on the structure of DHPS (Fig. 6A). The average values ranged from −2.921  (highly destabilising) to 0.182 kcal/mol (highly stabilising). The overall score of the impact of mutations ranged from −15 (highly stabilising mutations) to 17 (highly destabilising mutations). These scores were color-coded and depicted on the structure of DHPS (Fig. 6B). For clinically identified mutations reported in the literature, the mCSM predictions mostly indicate destabilising effects (Table 3). These effects are depicted on the structure (Fig. 6C).

### 3.4 RNAP (RNA Polymerase)

RNA Polymerase is an essential enzyme that mediates DNA-depended RNA synthesis in mycobacteria as in other organisms. The holoenzyme complex is a heterohexameric protein comprised of six chains (A, B, C, D, E, F) that are encoded by *rpoA, rpoA', rpoB, rpoC, rpoD, rpoZ, rpoT* genes in *M. leprae*. The model also contains the nucleic acid scaffold with non-template and template DNA, and a three-nucleotide stretch of an RNA transcript. This scaffold was borrowed from the template (PDB id: 5UHC) when modelling the complex for *M. leprae*. Mutations within the *rpoB and rpoC* genes are associated with resistance to rifampin, a bactericidal drug in the multi-drug therapy for leprosy. This heterohexameric model of RNA polymerase of *M. leprae* was modelled as published by us earlier [19]. Chain A was modelled from residues 3–226, chain B from 6 to 231, chain C from 28 to 1153, chain D from 3 to 1281, chain E from 28 to 108 and chain F from 253 to 574. Together, for 3259 residues, we generated 61,921 mutants. The predicted impacts of mutations on the stability of the complex and its affinity to rifampin, nucleic acid scaffold and subunit interfaces were computed for mutations in the entire structure unlike just the chain C (the beta-subunit of RNAP) as we published earlier. Effects of clinically identified mutations and their overall impact on the structure is shown in Table 4. For mCSM-lig, the value of zero indicates neutral impact and NA indicates the residue is located at a distance of more than 5 Å from rifampin.

**Table 4:** Predicted stability and affinity change for clinically identified mutations in chain C (beta subunit) of RNAP in rifampin-resistant *M. leprae* strains.

Mutations in beta subunit of RNAP	mCSM (ΔΔG in kcal/mol)	mCSM-lig (log change)	mCSM-NA (ΔΔG in kcal/mol)	mCSM-ppi (ΔΔG in kcal/mol)	DynaMut (ΔΔG in kcal/mol)	Overall Impact	Reference
A411T	−0.66	NA	3.68	−0.29	0.06	Moderate Impact	[50]
V424G	−1.48	NA	0.03	−0.50	−0.54	High Impact	[51]
G432S	−0.56	−0.83	3.68	0.48	1.17	Moderate Impact	[50]
T433I	−0.24	−0.57	−3.56	0.11	2.06	Moderate Impact	[50]
L436P	−1.19	−0.74	0.04	−0.70	−2.07	High Impact	[50]
Q438V	0.05	−0.94	−1.61	−0.21	1.4	Moderate Impact	[50]
D441V	1.67	−0.20	0.34	0.17	2.82	Moderate Impact	[50]
D441Y	0.17	−0.14	5.99	−0.26	1.12	Moderate Impact	[50]
D441N	−0.06	−0.10	2.00	−0.43	0.71	High Impact	[50]
Q442H	0.58	−0.12	1.77	0.36	3.44	Moderate Impact	[52]
N443S	0.18	0.06	2.13	−0.11	0.42	Moderate Impact	[53]
P445G	−2.16	0	0.03	−0.49	0.92	High Impact	[53]
P445A	−1.77	0	0.03	−0.49	2.01	Moderate Impact	[53]
L446V	−1.71	0	0.04	−0.81	−5.29	Moderate Impact	[53]
H451Y	−0.10	−0.09	2.31	−0.34	2.17	Moderate Impact	[50]
H451D	−1.73	−0.75	−3.63	−0.39	−3.03	High Impact	[50]
K452M	−0.28	−0.08	−2.93	−0.40	1.51	Moderate Impact	[53]
R453F	−1.90	−0.18	3.36	−0.32	−1.28	High Impact	[53]
S456L	−0.19	−0.30	−3.55	−0.07	3.12	Moderate Impact	[50]
S456M	−0.15	−0.36	−3.56	0.068	2.36	Moderate Impact	[50]
S456F	−0.83	−0.27	2.08	−0.29	6.85	Moderate Impact	[50]
S456W	−0.81	−0.45	4.92	−0.54	4.99	Moderate Impact	[50]
L458V	−1.11	−1.26	0.04	−0.32	−1.09	High Impact	[50]
G459A	−0.47	−0.71	0.06	−0.44	0.68	Moderate Impact	[53]
G461A	−0.28	NA	−0.04	0.39	−3.21	High Impact	[53]
S464W	−0.73	−0.63	4.53	−0.31	2.47	Moderate Impact	[53]
E466Q	0.44	−0.56	1.51	−1.04	−0.77	High Impact	[53]
G469L	−0.43	NA	−0.12	−0.91	−0.15	High Impact	[53]
G469P	−0.43	NA	−0.13	−0.84	−0.47	Moderate Impact	[53]
L470I	−0.71	NA	0.09	−0.19	0.66	Moderate Impact	[53]
E471K	0.31	NA	3.52	−0.15	−0.50	High Impact	[53]
R473G	−1.81	NA	−2.20	−0.51	−1.59	High Impact	[53]
V475M	−0.70	NA	0.04	−3.30	0.40	High Impact	[53]
H479N	−2.01	NA	−1.67	−1.37	−3.69	High Impact	[53]
G481R	−1.31	NA	2.40	−2.31	−1.44	High Impact	[53]
E487K	0.61	−0.22	3.32	−0.30	0.40	High Impact	[53]
P489L	−0.81	−0.25	−0.28	−0.95	1.23	High Impact	[53]
E490Q	−0.21	−0.58	1.85	−0.75	−1.14	High Impact	[53]
R505W	−0.25	NA	6.20	−0.56	2.47	Moderate Impact	[50]

*NA = Not applicable.

**Fig. 7:**
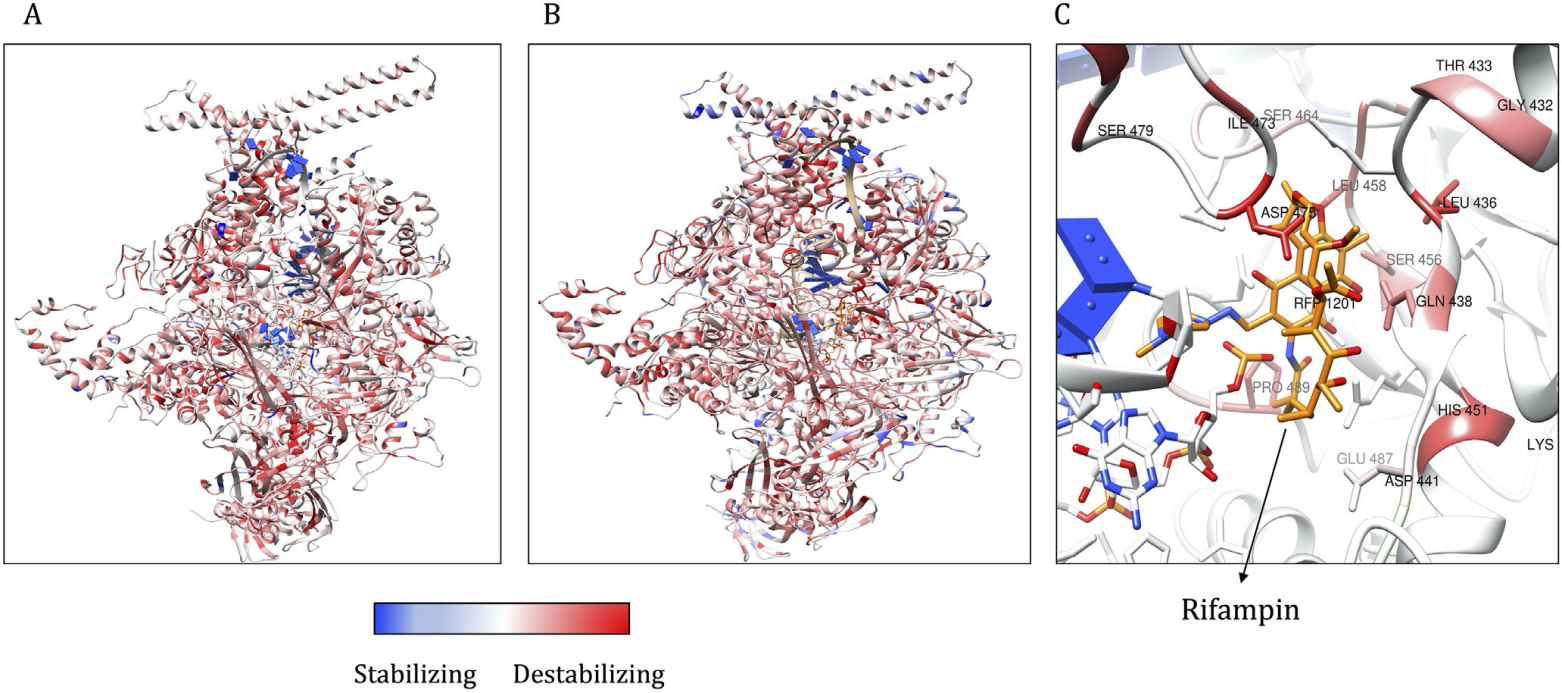
A: Average stability changes predicted by mCSM for all possible mutations in all the subunits of RNAP complex. These are depicted on the model and color-coded as red for values less than zero (destabilizing) and blue for values greater than zero (stabilizing). B. Average impact score for all possible mutations is depicted on the model of RNAP and color-coded as described in Fig. 7A. C. Stability changes as predicted by mCSM for mutations (stated in Table 4) in the rifampin binding site that are clinically resistant. Red color indicates average destabilizing effects at each residue position when mutated to all other amino acids. (For interpretation of the references to color in this figure legend, the reader is referred to the web version of this article.)

The average stability changes predicted by mCSM for systematic mutations at each residue position are computed and depicted on the model (Fig. 7A). The predicted ΔΔG values ranged from −4.312 (highly destabilising) to 2.716 (highly stabilising) kcal/mol. The overall scores for all the mutations ranged from −18 (highly stabilising) to 22 (highly destabilising) (Fig. 7B). Clinically identified mutations and stability changes that are predicted by mCSM were depicted on the model (Fig. 7C).

### 3.5 GYR (DNA Gyrase)

DNA Gyrase (GYR) is an essential enzyme in mycobacteria that catalyses ATP-dependent transient cleavage and negative supercoiling of closed circular DNA. The heterotetrameric protein (GyrA2, GyrB2) is comprised of four chains (A-D) that are encoded by *gyrA* (ML0006) and *gyrB* (ML0005) genes in *M. leprae*. Mutations within the *gyrA* gene are associated with resistance to ofloxacin, a second-line bactericidal drug in the treatment of leprosy. The chain A (GyrA) was modelled from residue positions 16–922, and after removal of a stretch of 420 amino acids (intein), the final residue sequence of chains A is numbered from 16 to 501 (residue number 131 in the model corresponds to 551 in the amino acid sequence). Chain B is modelled from residues 440–678. The double helical DNA scaffold was modelled by superimposition with the template.

**Fig. 8:**
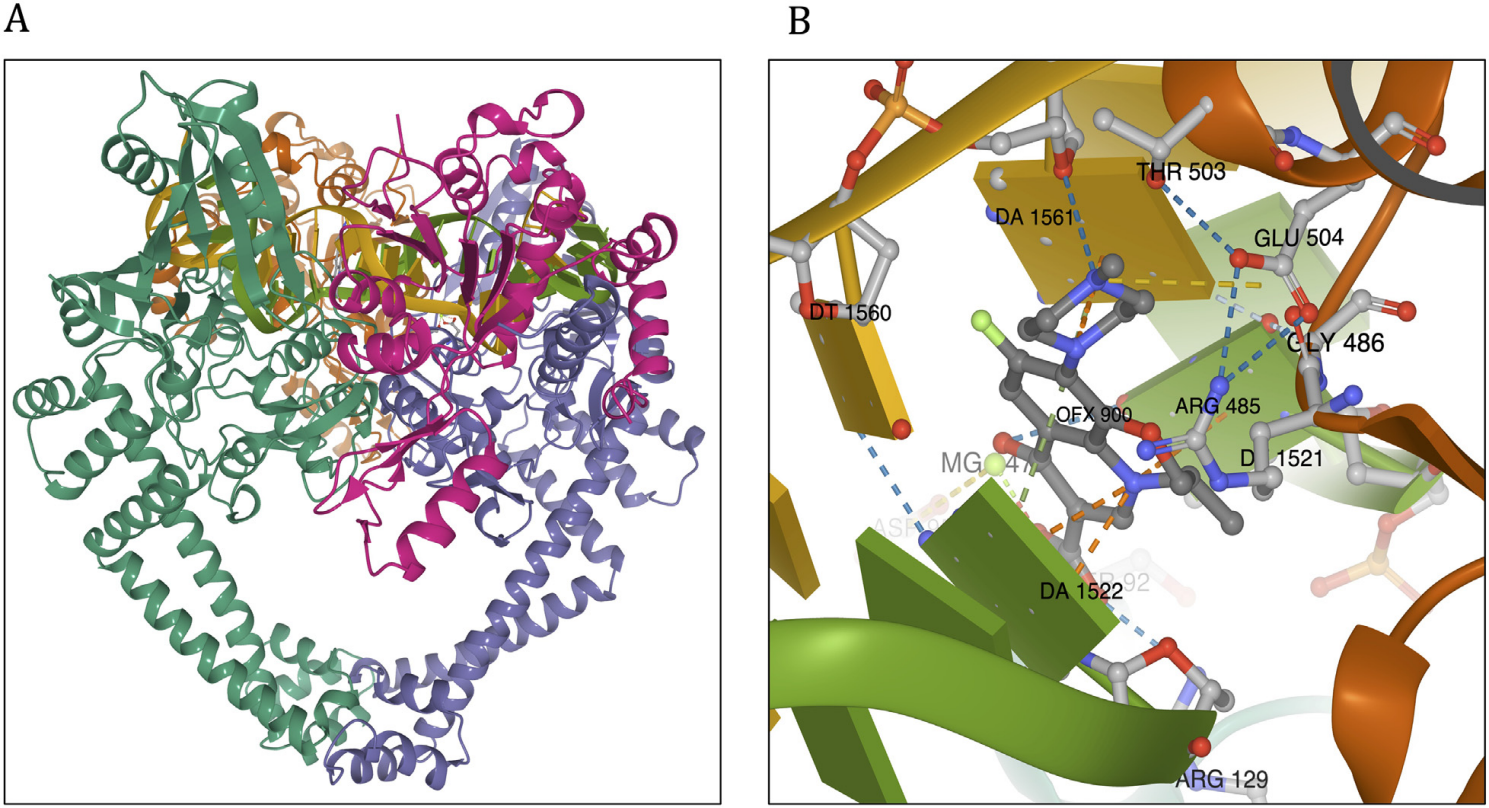
Model of DNA Gyrase (GYR) of *M. leprae*. A: Model of DNA Gyrase colored by chain id (chain A in green; chain B in orange; chain C in violet and chain D in magenta). B: Ofloxacin binding site and interatomic interactions of ofloxacin with the surrounding residue environment. The blue dotted lines indicate hydrogen bonds, the yellow dotted lines indicate ionic bonds, green dotted lines indicate pi-stacking, orange dotted lines are for cation-pi interactions, grey dotted lines represent hydrophobic interactions. (For interpretation of the references to color in this figure legend, the reader is referred to the web version of this article.)

The model was built with at least 100 iterations using Modeller v9.24, and the model with the lowest RMSD to the template (0.321 Å) was selected for further analysis. The resultant model has a Molprobity score of 0.86 at 100th percentile (this score is equivalent to the atomic resolution of the crystal structure). This model contains double-stranded DNA and a break in the strand at the active site for fluoroquinolone binding. The template has moxifloxacin in the active site at the interface between chain B and the DNA strand. We excised moxifloxacin from the model and introduced ofloxacin by molecular docking into the binding site using Glide XP module in Schrodinger Suite 2019-4 (Fig. 8).

Clinically identified mutations in the quinolone resistance determining region of the *gyrA* gene and corresponding amino acid substitutions are shown in Table 5. All the mutations indicate destabilising effects on the protein structure.

**Table 5:** Predicted stability and affinity change for clinically identified mutations in ofloxacin resistant *M. leprae* strains.

Mutations in GYR	mCSM (ΔΔG in kcal/mol)	Prime MM/GBSA (ΔΔG in kcal/mol)	mCSM-NA (ΔΔG in kcal/mol)	mCSM-PPI (ΔΔG in kcal/mol)	DynaMut (ΔΔG in kcal/mol)	Overall Impact	Reference
A91T	−0.70	−8.54	1.48	−0.28	−0.66	High Impact	[54]
A91V	−0.32	5.43	0.44	−0.59	−0.27	High Impact	[50]
S92A	−0.60	−12.35	−1.09	0.20	1.55	High Impact	[54]
R107L	−0.50	−6.33	−1.53	0.12	−0.26	Moderate Impact	[49]

**Fig. 9:**
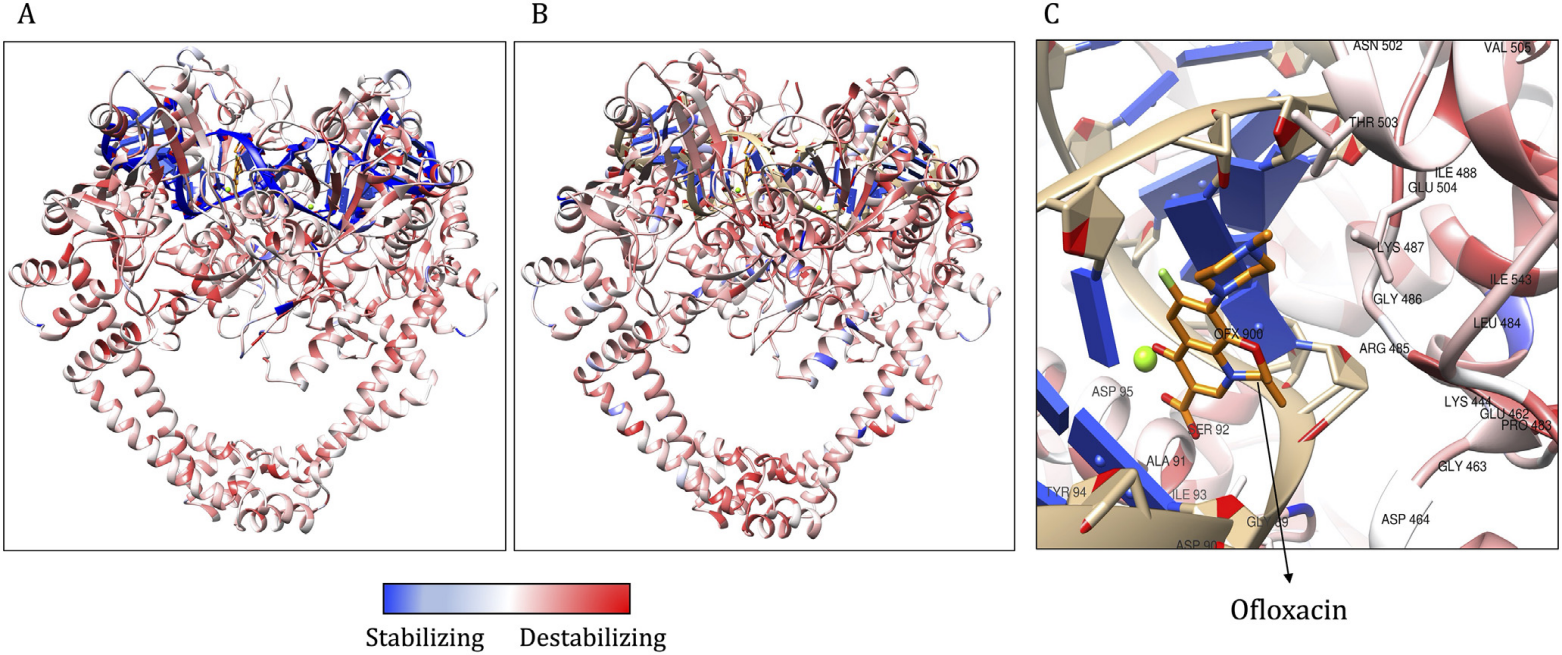
A: Average stability changes, as predicted by mCSM and depicted on the model. Regions in red indicate average destabilizing effects for all possible mutations at a specific residue position and blue indicate stabilizing effect. B. The average impact score mapped on the structure. C: Average of mCSM stability predictions for all possible mutations within the Ofloxacin binding site. Red color indicates that on an average, any mutation in this site induces a destabilizing effect on the protein. (For interpretation of the references to color in this figure legend, the reader is referred to the web version of this article.)

Average stability changes as predicted by mCSM at each residue position in GYR ranged from −4.256  (highly destabilising) to 2.036 kcal/mol (highly stabilising). The overall impact score for each mutation ranged from −11 (highly stabilising) to 21 (highly destabilising). These impacts were depicted on the model and colour-coded blue for stabilising and red for destabilising impacts (Fig. 9).

### 3.6 Clinically identified mutations

The mutations noted clinically in patient samples were largely confined to DRDR as the WHO recommended PCR protocol includes only the amplification and detection of mutations in DRDR to diagnose drug resistance in leprosy. As most of the DRDRs line the drug-binding sites of the target proteins, they have an impact on the ligand binding as noted by destabilizing effects (measured using mCSM-lig and Prime MM/GBSA) for most of the mutations (Table 3, Table 4, Table 5). From the predicted impacts, as shown in Supplementary Table S1, mutations that are highly detrimental to the stability of the drug target or have highly destabilizing impacts on the ligand binding are not identified clinically. This could be due to the fitness cost of these mutations to the bacteria [8]. These mutations are chosen based on the top five highly destabilizing effects on overall thermodynamic stability, protein-ligand, protein interfaces and protein-nucleic acids affinities.

## 4 Discussion

Quantifying the effects of point mutations on thermodynamic stability and function of the drug-targets in *M. leprae* provides mechanistic insights into the association between enthalpic changes and antimicrobial resistance phenotypes in leprosy. We present a publicly available web resource that provides predicted stability and affinity changes due to mutations in the drug targets for three major anti-leprosy drugs namely dapsone, rifampin and ofloxacin. Resistance has been noted for all the three drugs in leprosy endemic countries. In the absence of a rapid and confirmatory method to determine drug resistance in leprosy, clinicians and researchers rely on the presence/absence of mutations in drug-target coding genes as the proxy to diagnose drug resistance. These mutations are confirmed either by *in-vivo* experiments (by propagating mutant strains in the hind footpads of mice administered with anti-leprosy drugs) or by comparing the effects of the mutations in homologous genes of *M. tb.* A resource like HARP can help mycobacterial researchers to have an overview of the potential structural impacts of point mutations and the corresponding antimicrobial resistance outcomes in leprosy. The user can explore the structure, sequence driven and vibrational entropy-based stability changes for all possible mutations and understand their impact on the protein-ligand, protein-nucleic acid and protein-protein affinities.

Mutations that confer drug resistance in leprosy are usually identified in DRDR however, there are reports stating the occurrence of mutations beyond this region [49], [55]. Deciphering impacts of such mutations aid in better understanding of the allosteric mechanisms that drive resistance phenotypes. In HARP, by modelling mutations across the structure, we generated a resource that presents the impacts of not only known but new and emerging mutations associated with DHPS, RNAP and GYR in *M. leprae*. To our knowledge, HARP is one of its kind resources, developed exclusively for leprosy with comprehensive data related to predictions of stability and affinity changes for all possible mutations in the drug-targets. Other databases that document drug resistance in leprosy are MycoResistance [56] that provides a collection of studies reporting fluoroquinolone resistance, The Comprehensive Antibiotic Resistance Database (CARD) [57] that presents information on published reports related to resistant strains and their sequences, and recently, DRAGdb [58] that used PROVEAN to estimate the functional effects of reported resistance mutations in the sequences of *rpoB* and *gyrA* genes in *M. leprae.*

Computational saturation mutagenesis guides experimental approaches to study the impacts or help rationalise the consequences of known or emerging mutations [59]. Such approaches have been applied to other proteins like artificial (βα)_8_‐barrel protein [19] or in deep mutation scans [60]. Experimental validation of mutation impacts in *M. leprae* are time and labour-intensive processes owing to the inability of bacillus to grow on an artificial culture media. A resource like HARP can facilitate prioritisation of experiments and aid clinicians and researchers working in leprosy to have a quick and detailed perception of the possible impacts of the mutations in drug-resistant leprosy cases. We strongly believe that HARP will be highly beneficial to leprosy research.

## Declaration of Competing Interest

The authors declare that they have no known competing financial interests or personal relationships that could have appeared to influence the work reported in this paper.
