# Supplementary material for: HARP: a database of structural impacts of systematic missense mutations in drug targets of Mycobacterium leprae
Source: Comput Struct Biotechnol J. 2020 Nov 19;18:3692–704. doi: 10.1016/j.csbj.2020.11.013 (PMC7711215; doi:10.1016/j.csbj.2020.11.013)
Supplement: Supplementary data 1 [file mmc1.docx]

**Table 6:** Top five highly deleterious mutations predicted in each of the three drug-targets DHPS, RNAP and GYR. (NA = Not applicable as DHPS has no nucleic acids in the structure).

| Drug Target | Chain | Mutation | mCSM (ΔΔG in kcal/mol) | mCSM-lig (log change)  /Prime MM/GBSA (in kcal/mol) | mCSM-NA (ΔΔG in kcal/mol) | mCSM-ppi (ΔΔG in kcal/mol) | DynaMut (ΔΔG in kcal/mol) | Overall Impact |
| --- | --- | --- | --- | --- | --- | --- | --- | --- |
| DHPS | A | L12D | -3.37 | -36.52 | NA | -0.67 | -10.00 | High Impact |
| DHPS | A | L12G | -3.34 | -19.58 | NA | -0.34 | -10.61 | High Impact |
| DHPS | A | Y146G | -3.25 | -10.29 | NA | -1.01 | -6.95 | High Impact |
| DHPS | A | L12S | -3.18 | -24.09 | NA | -0.12 | -7.31 | High Impact |
| DHPS | A | L216G | -3.16 | -10.03 | NA | -0.49 | -10.53 | High Impact |
| RNAP | C | V176D | -3.03 | -0.74 | -0.24 | -0.53 | 0.11 | High Impact |
| RNAP | C | V176E | -2.88 | -0.75 | -0.23 | -0.66 | -1.22 | High Impact |
| RNAP | C | M440A | -2.79 | 0.17 | 0.03 | -0.51 | -7.66 | High Impact |
| RNAP | C | V176G | -2.75 | -0.22 | 0.02 | -0.29 | -5.62 | High Impact |
| RNAP | C | V176S | -2.66 | -0.34 | 3.66 | -0.16 | -7.44 | High Impact |
| GYR | A | I487S | -3.55 | -56.93 | 1.88 | -0.63 | -8.34 | High Impact |
| GYR | A | I487D | -3.48 | -6.20 | 1.89 | -0.77 | -10.00 | High Impact |
| GYR | A | I93D | -3.47 | -70.26 | 2.07 | -0.56 | -10.15 | High Impact |
| GYR | A | I487G | -3.44 | -7.07 | -0.31 | -0.63 | -10.14 | High Impact |
| GYR | A | L106D | -3.41 | -9.62 | 2.07 | -1.12 | -8.11 | High Impact |
